# Evidence is stronger than you think: a meta-analysis of vitamin C use in patients with sepsis

**DOI:** 10.1186/s13054-018-2191-x

**Published:** 2018-10-11

**Authors:** Jing Li

**Affiliations:** Santa Barbara Cottage Hospital, P.O. Box 689, Santa Barbara, CA 93102 USA

**Keywords:** Sepsis, Severe sepsis, Sepsis management, Vitamin C, Ascorbic acid, Meta-analysis

## Abstract

Two recent publications by Sheikh and Horner and Teng et al. reviewed studies on incorporating vitamin C to treat septic patients; however, a meta-analysis was not offered in either report. This commentary extends both reviews by integrating a meta-analysis and sharing aggregated results. Pooled analyses demonstrated a marked reduction in mortality and duration of vasopressor administration in the group with the use of vitamin C.

## Background

Sepsis is a severe condition with high mortality rates. Sheikh and Horner [1] and Teng et al. [2] recently published review articles regarding the incorporation of vitamin C in the treatment of sepsis. While the reviews offered insightful appraisals regarding the original studies and reported them in perspective, a meta-analysis was not produced and therefore aggregated quantitative results were not available for review. Considering both reviews were recent publications, this commentary extends their work by performing a meta-analysis and demonstrating the pooled results.

## Main text

Sheikh and Horner [1] conducted a literature search of EMBASE, Medline, and PubMed through January 2018 surrounding sepsis and intravenous vitamin C; Teng et al. [2] completed a review of the literature using PubMed in terms of sepsis and vitamin C. Teng et al. summarized five pertinent papers in their review; however, two of the original articles described in their review, one written by Fowler et al. [3] and the other by Natarajan et al. [4], were derived from the same clinical trial. In addition, the study reported by Tanaka et al. concerned patients with burns [5]. Although the authors reasoned that most deaths in modern burn centers are from septic shock [5], this paper was deemed irrelevant for the review and meta-analysis on sepsis and vitamin C. After removing one of the duplicate studies—the Natarajan et al. paper—and the Tanaka et al. paper, the three studies summarized by Teng et al. matched those of Sheikh and Horner. A search of EMBASE and PubMed similar to that of Sheikh and Horner was performed on 8 June 2018, but no further full-text articles were found; therefore, the three papers reviewed by Sheikh and Horner were included in the meta-analysis.

Table 1 describes the characteristics of the included studies and assesses the quality and potential biases of each study. Even though all three studies looked at treating septic patients with vitamin C, the quality of results varied due to differences in study design, inclusion criteria, and sample size. The studies by both Fowler et al. [3] and Zabet et al. [6] were randomized, double-blind, placebo-controlled clinical trials of small sample sizes, while the Marik et al. study [7] was a before-after study with propensity score adjustment. It is worth noting that Marik et al. included intravenous hydrocortisone and thiamine, in addition to vitamin C, thereby introducing confounders into the study [7]. Three clinical outcomes were reported by at least two articles, which were mortality, intensive care unit length of stay (ICU-LOS), and vasopressor duration [3, 6, 7].

**Table 1:** Characteristics of included studies

Study	Year	Design	*n*	Inclusion criteria	Exclusion criteria	Participant characteristics	Dose of vitamin C	Outcomes reported	Quality assessment
Marik et al. [7]	2017	Retrospective before-after study	94	Primary diagnosis of severe sepsis or septic shock and a procalcitonin level ≥ 2 ng/mL	< 18 years, pregnant, or with limitations of care	Mean age for treated group 58.3 years, 57% male; mean age for control group 62.2 years, 49% male	1.5 g IV every 6 h	Hospital mortality, ICU-LOS, duration of vasopressors, RRT for AKI, reduction in serum procalcitonin and SOFA over the first 72 h	Neither randomized nor blinded, although propensity-adjusted; protocol included intravenous hydrocortisone and thiamine, in addition to vitamin C
Fowler et al. [3]	2014	Prospective phase I trial	24	Diagnosis of severe sepsis	< 18 years, pregnant, prisoners, cognitively impaired and unable to provide consent, or non-English speakers	Age for treated group: 30–92 years, 56% male; age for control group: 54–68 years, 50% male	50 mg/kg/day, or 200 mg/kg/day	Vitamin C safety and tolerability, days on vasopressor, ventilator-free days, ICU-LOS, 28-day mortality	Randomized, double-blind, placebo-controlled, but underpowered
Zabet et al. [6]	2016	Prospective clinical trial	28	Adult (18–65 years) with diagnosis of septic shock and required vasopressor drug to maintain MAP > 65 mmHg		Mean age for treated group 64.14 years, 71% male; mean age for control group 63.71 years, 79% male	25 mg/kg IV every 6 h	Vasopressor dose and duration, ICU-LOS, 28-day mortality	Randomized, double-blind, placebo-controlled, but small sample size

*AKI* acute kidney injury, *ICU-LOS* intensive care unit length of stay, *IV* intravenous, *MAP* mean arterial pressure, *RRT* renal replacement therapy, *SOFA* Sepsis-Related Organ Failure Assessment

A meta-analysis was performed on these three outcomes using Comprehensive Meta-Analysis (version 3.3.070). Considering diversity in the study populations and differences in the treatments including varying doses of vitamin C, a random-effects model was used in all analyses. Mortality was considered the primary outcome for this meta-analysis, and a fail-safe N test was carried out to assess publication bias. Fowler et al. randomized patients into three groups: low-dose ascorbic acid, high-dose ascorbic acid, and placebo [3]. The low-dose and high-dose groups were combined into the vitamin C group for meta-analysis. If the original study did not incorporate a power analysis or mention a one-sided or two-sided test, a two-sided test was assumed.

**Fig. 1:**
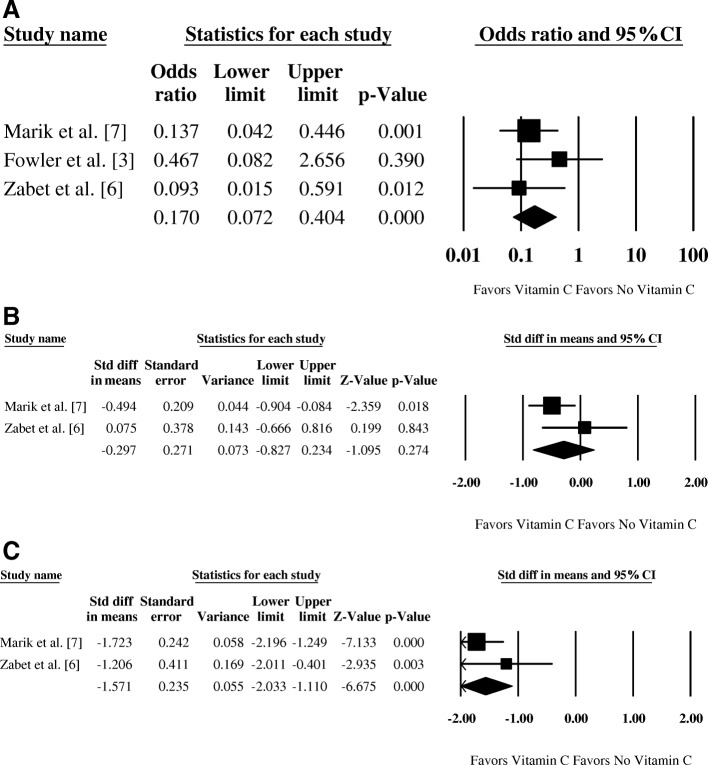
Pooled analyses of mortality (**a**), ICU-LOS in days (**b**), and vasopressor duration in hours (**c**). ICU-LOS intensive care unit length of stay, CI confidence interval

All three studies reported mortality rates between the experimental arm with the use of vitamin C and the control arm without vitamin C. While the mortality results from two studies [6, 7] favored the vitamin C treatment at the significance level of 0.05, the study by Fowler et al. [3] did not reach statistical significance. Pooled analysis of all three studies revealed a marked reduction in mortality with the use of vitamin C (odds ratio (OR) = 0.17, 95% confidence interval (CI) 0.07–0.40; *p* < 0.001; Fig. 1a). No significant heterogeneity between studies was found (*I*^2^ = 0; *p* = 0.40 for Cochran’s Q). Due to the strong effect size of two of the three studies, the computed fail-safe N would require nine null-finding studies to render this pooled result nonsignificant at α = 0.05.

All three studies reported ICU-LOS. Results from Marik et al. [7] and Zabet et al. [6] were incorporated in the meta-analysis; however, the study by Fowler et al. [3] was excluded as it did not provide either standard deviation (SD) for direct synthesis or the median for estimating SD [8]. Although Marik et al. reported median and interquartile range for ICU-LOS in their publication, the mean and SD were supplied by Dr. Marik via an email inquiry [7]. Pooled analysis favored the use of vitamin C, but statistical significance was not reached (standardized mean difference (SMD) = −0.30, 95% CI −0.83 to 0.23; *p* = 0.27; Fig. 1b). Heterogeneity between these two studies was not significant (*I*^2^ = 42.3%; *p* = 0.19 for Cochran’s Q).

All three studies reported the duration of vasopressor use. Results from Marik et al. [7] and Zabet et al. [6] were incorporated in the meta-analysis; however, the study by Fowler et al. [3] was excluded as it did not provide either SD for direct synthesis or the median for estimating SD, and the duration was summarized in days while the other two studies reported hours [8]. Pooled analysis showed a significant reduction in duration of vasopressor administration in the group with vitamin C (SMD = −1.57, 95% CI −2.03 to −1.11; *p* < 0.001; Fig. 1c). Heterogeneity between these two studies was not significant (*I*^2^ = 15.0%; *p* = 0.28 for Cochran’s Q).

## Conclusions

Despite varying degrees of statistical significance between the original studies, this meta-analysis reveals a positive correlation between incorporating vitamin C in the treatment of sepsis and favorable patient outcomes, including better survival and shorter duration of vasopressor use; *I*^2^ was shown to be insignificant, and therefore corroborates the consistency of evidence. Since this is a quantitative synthesis of a small number of studies, further randomized clinical trials are required to prove a causal relationship. If this relationship is confirmed, vitamin C has enormous potential to improve patient care and reduce mortality rates due to its low cost and wide availability.

## Abbreviations

ICU-LOS: Intensive care unit length of stay
CI: Confidence interval
OR: Odds ratio
SD: Standard deviation
SMD: Standardized mean difference
